# Biosafety and Biosecurity: A Relative Risk-Based Framework for Safer, More Secure, and Sustainable Laboratory Capacity Building

**DOI:** 10.3389/fpubh.2015.00241

**Published:** 2015-10-21

**Authors:** Petra Dickmann, Heather Sheeley, Nigel Lightfoot

**Affiliations:** ^1^dickmann risk communication drc, London, UK; ^2^Public Health England (PHE), London, UK; ^3^Connecting Organizations for Regional Disease Surveillance (CORDS), Lyon, France; ^4^Chatham House, Centre on Global Health Security, London, UK

**Keywords:** biosafety, biosecurity, laboratory capacity building, biorisk, international health regulations, emerging infectious diseases

## Abstract

**Background:**

Laboratory capacity building is characterized by a paradox between endemicity and resources: countries with high endemicity of pathogenic agents often have low and intermittent resources (water, electricity) and capacities (laboratories, trained staff, adequate regulations). Meanwhile, countries with low endemicity of pathogenic agents often have high-containment facilities with costly infrastructure and maintenance governed by regulations. The common practice of exporting high biocontainment facilities and standards is not sustainable and concerns about biosafety and biosecurity require careful consideration.

**Methods:**

A group at Chatham House developed a draft conceptual framework for safer, more secure, and sustainable laboratory capacity building.

**Results:**

The draft generic framework is guided by the phrase “LOCAL – PEOPLE – MAKE SENSE” that represents three major principles: capacity building according to local needs (local) with an emphasis on relationship and trust building (people) and continuous outcome and impact measurement (make sense).

**Conclusion:**

This draft generic framework can serve as a blueprint for international policy decision-making on improving biosafety and biosecurity in laboratory capacity building, but requires more testing and detailing development.

## Introduction

International prevention and control of infectious diseases requires a variety of health (human and animal) system capacities, including laboratory capacity for surveillance and diagnostics worldwide (1–3). This capacity building is recognized and enforced by the revised International Health Regulations (IHR 2005) of the World Health Organization (WHO) (4–6). Building laboratory capacity worldwide is, however, an activity with intrinsic complexities and paradoxes: countries with high endemicity of disease often have low resources (water, electricity) and capacities (laboratories, trained staff, adequate regulations) to survey and diagnose the diseases caused by these agents, whereas countries with low endemicity of high pathogenic agents often have high-containment facilities with costly infrastructure and maintenance.

With increasing laboratory capacity worldwide the global health security agenda considers biosafety and biosecurity topics as relevant (7–9). In the international convention, *Biosafety* refers to “principles, technologies, practices, and measures implemented to prevent the accidental release of, or unintentional exposure to pathogenic agents” (10). *Biosecurity* refers to the “protection, control, and accountability measures implemented to prevent the loss, theft, misuse, diversion, or intentional release of pathogenic agents and related resources as well as unauthorized access to, retention or transfer of such material” (10). It is necessary to get the balance of concerns over proliferation and the need to diagnosis and undertake surveillance.

Biomaterials have to be handled safely and securely in all settings: work on harmful and infectious biological materials inside laboratories needs to be contained and separated from a not-contaminated environment (biocontainment). To ensure the safety of laboratory personnel and the environment, work on pathogens in high-resource laboratory settings is classified in biosafety levels 1–4 (BSL1–4) (11). This classification draws on the concept of step-wise biocontainment that aims at keeping the pathogen confined to a designated space. Biocontainment, however, allows for a relative risk based, differential approach by focusing on the difference – the barrier – between a pathogenic interior and a less pathogenic exterior. The physical containment of pathogens protects the workers or the unintended release of pathogens that could lead to laboratories being the source of outbreaks. In high-resource settings, regulations for safe operations in animal and human health laboratories exist (12–16). However, despite its containment standards laboratory accidents (17), unintentional exposure (18–20), and unintentional release (21, 22) occur and maintenance of these containment standard are strongly recommended (23, 24).

Additionally, security concerns exist about the proliferation of high-containment laboratories and the illegal acquisition or intentional release of high consequence pathogenic agents. Systems designed to prevent the misuse of potentially hazardous biological material are generally described as biosecurity systems. Despite its importance, biosecurity concepts and approaches, however, are less well understood, not always adequately reflected, connected, and implemented with biosafety systems (25, 26). Recently, a framework to help evaluate biosafety and biosecurity incidents provides a useful rationale (27). This framework uses a seven category scale to assess incidents and situation in this regards to their impacts on personnel, integrity of containment, and the environment (27). Using this metric and rating system for risk assessment could improve the common understanding of biosafety and biosecurity risks and facilitate risk communication.

To ensure the safe and secure handling of pathogenic agents the current practice is mainly focused on exporting the complex costly containment, “western standard” of laboratory safety into vulnerable areas of the world with high demand for laboratory activity due to emergency outbreak situations or a continuous high prevalence. This practice has not always been effective, to date, needed investments have not been fully sustainable, the “western standard” of biosafety relies on adequate and continuous access to resources and educated personnel (28–30). Biosecurity concepts and approaches have been very slow to be addressed this at the same rate as the development of biosafety systems (31).

The Global Health Security Agenda points to a lack of collaboration and integration of health and security approaches and communities and thus calls to end the silo-thinking and encourages an integrated health security approach (5, 32–34). There is a need for new and innovative approaches to building laboratory capacity worldwide, and in particular in low-resource settings, and ensure safe and secure handling of biomaterials.

### Safe and Secure Biomaterial Project

To this end, a Chatham House project on “Safe and Secure Biomaterials,” funded by the UK International Biosecurity Programme, aimed to explore and analyze the practice and regulations of biosafety and biosecurity in low- and high-resource settings and develop a generic framework that could guide international policy decision-making to build laboratory capacity with a focus on low-resource settings.

This project had two phases. In phase 1 (2012) of this research project, stakeholders were convened to discuss the research problem, the project plan of international laboratory capacity building and to provide an overview of the landscape of biosafety and biosecurity regulations and guidelines. In a Chatham House research paper, the outcomes of the first phase describes the need for laboratory capacity building, the discrepancy between endemicity and resources, different standards of biosafety and biosecurity regulations in G7 and seven low- and middle-income countries and the isolated approaches of the health and security communities (35, 36). The research paper suggests a relative risk approach to rethink current regulations and practices for further exploration in the next phase of the project (37, 38).

Phase 2 of the project (2014) used the conceptual framing of the relative risk approach to discuss and develop a generic framework for a safer, more secure, and sustainable laboratory capacity building. In a scoping meeting with international stakeholders, the approach and initial framework ideas were discussed and tested to prepare for a broader workshop. This scoping meeting was attended by 17 senior experts from low-, middle-, and high-income countries, international organizations, and research institutions.

The bigger workshop offered a forum for intense collaboration and interaction of different stakeholders from low- and high-resource settings; the bigger workshop was attended by 23 senior experts from biomedical sciences, engineering, and policy-making from both fields of biosafety and biosecurity in low-, middle-, and high-income countries. In this bottom-up approach, the main pillars of the generic framework were created. This workshop also serves to agree on recommendations and promote this framework for adoption into the international discourse of biosafety and biosecurity.

This article describes the generic framework, discusses the potential implication of this approach and summarizes the recommendations for a safer, more secure, and sustainable laboratory capacity building.

## Methods

Key and diverse stakeholders from differing disciplines and backgrounds reflected and determined a generic framework for assessing best solutions for laboratory capacity building (smaller scoping meeting; larger workshop). This was done in a two-step process: in a smaller scoping meeting, stakeholders were consulted to generate feedback on initial ideas to challenge conventional thinking and receive consensus regarding the relative-risk approach that would serve as a narrative to develop a generic framework in a broader workshop scenario.

In a second step, the workshop applied a creative, interactive approach to jointly reflect and elaborate on safer, more secure, and sustainable solutions for laboratory capacity building. The 2-day workshop was based on interactive, interdisciplinary work in small groups of people coming from different professional backgrounds (biomedical, sciences, engineering, and policy-making) with experience from both fields of biosafety and biosecurity in low-, middle-, and high-resource settings^1^. The workshop applied a focus group approach and emphasized group work that was structured to trigger dialog and debate on current practices, stimulate thinking about what changes could lead to safer, more secure, and sustainable laboratory capacity building (particularly in low-income countries), and capture participant insights on the specific components, attributes, and principles of such changes. People from a diverse background and experience used analytical tools and templates to analyze the contradictions, contrasts, and issues and presented their results in plenary sessions for broader discussion.

## Results

### Relative-Risk Approach

The relative-risk approach to safe and secure laboratory capacity building moves away from the use of predetermined standards under which work on particular pathogens should be performed. Such standards were felt to represent a Western and high-resource setting perspective that is far from realities in different parts of the world and neither achievable nor sustainable in low-resource settings. The relative-risk approach focuses on conditions of biosafety and biosecurity that can make work safer, more secure, and sustainable in specific environments. To this end, the approach builds on a *contextual assessment of risks* and considers the system and environments (e.g., information, communication, and coordination systems) in which laboratory capacity is being built. This differential approach reflects and relates “inside” of the laboratory with “outside” the laboratory and considers the permeability of the barrier separating them. This approach focuses on the barrier that separates the inside from the outside and provides a structured assessment of the relative risks. Rather than defining the endpoint as adherence to Western standards, it focuses on agreed outcomes (e.g., maintaining the biocontainment barrier) and develops contextually appropriate, relevant parameters for biosafety and biosecurity.

Tools and templates used during the workshop incorporated this structure to help participants elicits the contextual parameters. The matrix templates were designed to reflect three dimensions:

- Inside: people
- Barrier: integrity of containment systems
- Outside: environment, e.g., structures and services

For each dimension (i.e., people, integrity of containment, structures, and services), groups identified key influencing factors and described how these could support innovative and sustainable solutions for a safer and more secure handling of biomaterials. To enable this discussion, the matrix required participants to reflect on an ideal situation and compare this to realities in high-resource and low-resource settings.

### Biosafety–Biosecurity

Biosafety and biosecurity requirements and concerns are often addressed separately and from different stakeholders’ perspectives. However, in this workshop, participants articulated a shift in the conventional understanding of biosafety and biosecurity: rather than assuming biosafety and biosecurity as different and discrete entities, participants stressed that biosafety and biosecurity aspects are often intertwined and interlinking. Participants agreed that the many aspects and activities of biosafety and biosecurity could be and should be woven together as a tapestry with multiple layers and components, and concluded that the two areas of concern overlap. For instance, safe operations in laboratories require trust between staff members, training, and reliable systems. The same aspects are relevant for biosecurity. Instead of stressing the differences, participants reinforced the approach to integrate biosafety and biosecurity in one interdisciplinary discourse.

### Objectives of a Generic Framework: SAFER

The main objectives for a generic framework for safer, more secure, and sustainable laboratory capacity building was captured by the SAFER acronym. SAFER stands for

- *S*ustainable: laboratory capacity can be maintained independently over a long-term period;
- *A*ffordable: communities do not depend on external aid for core functionality;
- *F*unctional: the laboratory staff are safer than currently, communities are at less risk of the laboratory being the source of the infection and the presence of the surveillance and diagnostic capability keeps the community safer;
- *E*ffective: laboratory capacity building presents the most suitable application for the specific environment; and
- *R*ealistic: laboratory capacity building is an answer to a question that the community actually has.

### Generic Framework: Local – People – Make Sense

The generic framework for sustainable, safer, and more secure laboratory capacity has three principles that are summarized in the phrase “LOCAL – PEOPLE – MAKE SENSE.”

The first key principle is the LOCAL principle. This includes

- Involving local people at every stage of capacity building;
- Building on existing infrastructure (assets and needs, on-going developmental plans and activities); and
- Meeting local requirements.
- Sustained locally

The second principle in laboratory capacity building is the PEOPLE principle. It is important to:

- Build relationships;
- Develop trust;
- Develop relevant skills (e.g., laboratory, construction, testing, etc.);
- Engage in two-way communication: gather intelligence insights on perceptions, culture, attitudes, and behaviors that then inform communication and compliance;
- Create networks; and
- Advocate for political buy-in.

The third principle reflects the need to monitor, measure, and evaluate the process of improving biosafety and biosecurity in support of necessary laboratory procedures. Making sense is an active process that includes

- Developing metrics to use in measurement and evaluation;
- Identifying relevance of settings and needs;
- Measuring starting points and progress;
- Evaluating effectiveness of the biosafety and biosecurity solution and networks of supporting structures of laboratory capability; and
- Learning and informing developmental planning.

### Capacity Building Action Areas: 4M

The key biosafety and biosecurity capacity building areas are summarized in the mnemonic 4M. These are

- HU*M*AN: referring to training, education, and communication activities;
- *M*ETHODS: referring to developing standard operating procedures and guidelines;
- *M*ATERIAL: referring to designing appropriate buildings (engineering and architecture), and security; and
- *M*ONEY: referring to advocating for resource allocations and developing a range of funding models.

### Framework to Guide Decision-Making

The framework is a matrix that applies the three key principles (left column) as a *y*-axis to the four capacity building areas (4M) on an *x*-axis (Table 1).

**Table 1:** **Local people make sense – key criteria for safer, more secure, and sustainable capacity building**.

	HU*M*ANS	*M*ETHODS	*M*ATERIAL	*M*ONEY
LOCAL				
PEOPLE				
MAKE SENSE				

The three key principles (Local – People – Make sense) should be applied to each of the capacity building areas. When considering capacity building, e.g., in the field of “HU*M*ANS,” training, education, and communication activities have to match the “Local People Make sense” criteria. Training has to include local people, building on existing infrastructures, and meet local requirements. Training objectives need to include relationship building, trust development among relevant skill building, etc. Training efforts should be monitored and measured against the SAFER objectives; this requires the development of an appropriate metric in context sensitive and variable environment, etc.

For capacity building in fields of methods, material, and money, the same procedure is envisioned: capacity building activities in the fields of methods, material, and money need to comply the Local – People – Make Sense principle.

The fields of capacity building activities (Humans, Methods, Materials, Money), as a *x*-axis, and the Local – People – Make Sense-principle, as a *y*-axis, presents the guiding frame for the concept and design of activities, the empty fields in the matrix give room to develop solutions in different contexts that make sense in diverse and changing environments.

## Discussion

### Generic Application for Local Solutions

This framework for sustainable, safer, and more secure laboratory capacity is generic and aims to guide decision-making. It does not provide answers, but helps to develop local, context-sensitive solutions. The application of this framework is seen as an iterative process. When facing decisions on if and how to improve laboratory capacity, the framework can help decision makers, scientists, architects, and other stakeholders to agree on and achieve SAFER outcomes.

### Framework for Planning, Monitoring and Evaluation

This matrix serves as a the core part of the conceptual framework that helps guide the (i) planning, (ii) monitoring, and (iii) evaluation of alternative approaches to laboratory capacity building worldwide. Although this framework is still in the drafting phase, participants felt that it indicates the right approach and provides planners, public and veterinary health scientists, policy-makers, engineers, architects, and other professionals with a useful tool.

### Global Initiatives with Local Solutions

Training and capacity building are well-established initiatives on the international agenda in development, science, and science. However, in contrast to many capacity building activities, this framework calls for a different approach: rather than implementation “western” goal and training programs it calls for sustainable training that has to include local people from the beginning in the design period, building on existing infrastructures and meet local requirements.

## Limitations

### Lack of Specific Guidelines

Among the limitations is, however, that this framework requires thinking. It is not applying ticks to pre-set check lists. Its generic nature can be criticized for being non-specific. However, this framework acknowledges that different disciplinary groups (engineers, architects, etc.) have created innovative technical solutions to discrete problems. This framework, however, has a more comprehensive and integrative understanding of biosafety and biosecurity; these concerns cannot be reduced and discussed on a level of isolated technical solutions. For instance, having naturally ventilated labs seem to be a good solution for an environment that has only intermittent access to power and constant climate conditions in laboratories, but a focus on individual technical solutions, such as natural ventilated labs, does not offer solutions for broader and more complex biosafety and biosecurity. These complex concerns require a different angle.

### Hypothetical Pilot Status of the Framework

This framework is a theoretical, pilot framework, and thus a draft. It was generated through a thorough analytic and reflective process involving key stakeholders. However, we believe that the application of the framework and the solutions generated with it in the future will add relevant feedback and probably amend and/or refinements.

## Recommendations

This draft framework will be further developed and contextualized. Envisioned are three major steps:

(1) Meetings to *populate* the framework
  - Technical meetings and implementation discussions with technical experts (engineers, architects, scientists, etc.) to *populate* the framework;
(2) Briefings to *popularize* the approach;
  - Briefings and discussion with key policy-makers;
  - Further support for countries to take this framework to political fora, international organizations, and working groups, such as World Health Assembly (WHA), the Global Health Security Agenda, and the Global Partnership to gain universal acceptance and support for such an approach.
(3) In a next step, this further refined framework should *promote* political buy-in and advocate for changes in the laboratory capacity building policy and in funding schemes.

## Conclusion

The common practice of exporting costly and complex biocontainment facilities and standards is not sustainable. Concerns about sustained biosafety and proliferation of capabilities that could be misused require careful consideration. The proposed alternative framework for a safer, more secure, and sustainable laboratory capacity building can guide capacity building according to local needs (local) with an emphasis on relationship and trust-building (people) and continuous outcome and impact measurement (make sense). This generic framework can and should serve as a blueprint for international policy decision-making on improving biosafety and biosecurity in laboratory capacity building.

## Author Contributions

PD developed the idea and material for the workshops and wrote the draft manuscript. HS and NL contributed to the workshop and draft manuscript. All authors approve the final version.

## Conflict of Interest Statement

The authors declare that the research was conducted in the absence of any commercial or financial relationships that could be construed as a potential conflict of interest.

## Footnotes

^1^Participants from low-, mid-, and high-income countries were chosen based on their experience in biosafety and biosecurity. The workshop was held under the Chatham House Rule; thus, we are not permitted to disclose names.
